# Characteristics of intestinal microbiota in C57BL/6 mice with non-alcoholic fatty liver induced by high-fat diet

**DOI:** 10.3389/fmicb.2022.1051200

**Published:** 2022-12-22

**Authors:** Guangwen Yan, Shuaibing Li, Yuhang Wen, Yadan Luo, Jingrong Huang, Baoting Chen, Shuya Lv, Lang Chen, Lvqin He, Manli He, Qian Yang, Zehui Yu, Wudian Xiao, Yong Tang, Weiyao Li, Jianhong Han, Fangfang Zhao, Shumin Yu, Fang Kong, Benazir Abbasi, Hongmei Yin, Congwei Gu

**Affiliations:** ^1^College of Animal Science, Xichang University, Xichang, China; ^2^Laboratory Animal Centre, Southwest Medical University, Luzhou, China; ^3^Model Animal and Human Disease Research of Luzhou Key Laboratory, Luzhou, China; ^4^State Key Laboratory of Quality Research in Chinese Medicine, Macau University of Science and Technology, Taipa, Macao SAR, China; ^5^College of Veterinary Medicine, Sichuan Agricultural University, Chengdu, China; ^6^College of Veterinary Medicine, Nanjing Agricultural University, Jiangsu, China

**Keywords:** non-alcoholic fatty liver disease, intestinal microbiota, 16S rDNA sequencing, mice, high-fat diet

## Abstract

**Introduction:**

As a representation of the gut microbiota, fecal and cecal samples are most often used in human and animal studies, including in non-alcoholic fatty liver disease (NAFLD) research. However, due to the regional structure and function of intestinal microbiota, whether it is representative to use cecal or fecal contents to study intestinal microbiota in the study of NAFLD remains to be shown.

**Methods:**

The NAFLD mouse model was established by high-fat diet induction, and the contents of the jejunum, ileum, cecum, and colon (formed fecal balls) were collected for 16S rRNA gene analysis.

**Results:**

Compared with normal mice, the diversity and the relative abundance of major bacteria and functional genes of the ileum, cecum and colon were significantly changed, but not in the jejunum. In NAFLD mice, the variation characteristics of microbiota in the cecum and colon (feces) were similar. However, the variation characteristics of intestinal microbiota in the ileum and large intestine segments (cecum and colon) were quite different.

**Discussion:**

Therefore, the study results of cecal and colonic (fecal) microbiota cannot completely represent the results of jejunal and ileal microbiota.

## Introduction

Recently, non-alcoholic fatty liver disease (NAFLD) has become the most common liver disease. In China, NAFLD has replaced Viral Hepatitis B as the most common chronic liver disease (Xiao et al., 2019). In the United States, NAFLD has become one of the main causes of liver transplantation (Gadiparthi et al., 2020). The gut microbiota recently emerged as a pivotal transducer of environmental influences (i.e., dietary components and drug treatments) to exert protective or detrimental effects on several host tissues and systems, such as the regulation of intermediary metabolism, liver function, and cardiovascular disorders, either directly *via* translocation or indirectly through microbial metabolism or process in metabolic disorders (Lim et al., 2019).

Current research has demonstrated that the gut microbiota structure in patients with fatty liver was significantly different (Aron-Wisnewsky et al., 2020). A study of children reported that the phyla *Bacteroidetes* and *Proteobacteria* were higher in NAFLD, whereas the phylum **Firmicute*s* were higher in children who were simply overweight or obese (Schwimmer et al., 2019). It is believed that these changes in intestinal microbiota result from diet and the body (Gao et al., 2020), and changes in the gut microbiota can cause changes in the body’s metabolic state (Xu et al., 2020). The liver and the intestine are tightly linked through portal circulation. Consequently, gut microbial-derived products arriving at the liver may have pathogenic implications (Abdou et al., 2016). In recent years, the role of the gut microbiota has been increasingly implicated in modulating risk factors for NAFLD, such as energy homeostasis dysregulation, insulin resistance, increase in intestinal permeability, endogenous production of ethanol, inflammation (innate immunity and inflammasomes), and choline and bile acid (BA) metabolism (He et al., 2016). These factors likely influence the pathogenesis of NAFLD (Aragonès et al., 2019). Gut microbiota-derived factors and short-chain fatty acids (e.g., acetate, propionate, butyrate) can have anti-inflammatory properties, which could prevent the progression of NAFLD, however lactate, ethanol, and trimethyl N-oxide (TMAO) derived from intestinal microbiota can induce a decrease in total bile acid pool size, which in turn can affect Farnesoid X receptor (FXR) signaling and NAFLD (Aron-Wisnewsky et al., 2020).

Currently, feces are the main sample type that is used for gut microbiome research in humans and large animals, and the cecum and feces are the common detection sites in small animals. However, the function and flora of each intestinal segment are different. A major disadvantage of using stool samples to determine the composition of gut microbes is that the fecal microbiota represents only the end of the colon. At the same time, other parts of the gastrointestinal tract, particularly the small intestine, are poorly studied. In research involving NAFLD, the jejunum and ileum microflora were less studied. The small intestine is a hostile environment for microbes to survive due to its short transport time, short digestion enzyme, and bile excretion time. It, therefore, requires a different survival strategy than the colon microbes (Zoetendal et al., 2012). Most microbial species and functions in the fecal community come from coliform bacteria, while only a few microbes are from the small intestine (Chambers et al., 2019). As mentioned above, based on the niche-specific colonization pattern of exogenous flora, donor E. coli microbes may prefer to colonize in the small intestine rather than the large intestine (Li N. et al., 2020). Therefore, whether it is representative to use cecal or fecal (colonic) contents to study intestinal microbiota in the study of NAFLD remains to be shown. In this study, the structural characteristics of intestinal flora in the jejunum, ileum, cecum, and colon (feces) of NAFLD mice were studied to analyze the validity of the cecum and colon as the main sites of intestinal flora research in the pathological state of non-alcoholic fatty liver disease.

## Materials and methods

### Animal study

#### Animal feeding and sample collection

Six-week-old specific pathogen-free male C57BL/6 mice (weighting 17–19 g) were purchased from Beijing Weitong Lihua Laboratory Animal Technology Co., LTD and housed at 22 ± 2°C and 50–60% relative humidity in a specific pathogen-free facility maintained on a 12-h light/dark cycle in the Laboratory Animal Center of Southwest Medical University. After 1 week of acclimatization, 20 mice were randomly divided into two groups for 16 weeks: Control (CK) group (*n* = 10, Normal diet, ND) and NAFLD (NA) group (*n* = 10, High fat diet, HFD). The normal diet comprised 65.08 kcal% carbohydrates, 23.07 kcal% proteins, and 11.85 kcal% fats, purchased from HUANYU BIO, while the HFD diet contained 20 kcal% carbohydrates, 20 kcal% proteins, and 60 kcal% fats, which was purchased from Research Diets. The composition of the normal and HFD diet is detailed in Supplementary Table S1. All experimental mice had free access to food and water. The physical activity, consumption of food and water, and excretion of experimental mice were observed daily.

At the end of the prescribed feeding period, all mice were fasted overnight and anesthetized with an intraperitoneal injection of 1% pentobarbital sodium (50 mg/kg body weight). After anesthetization, blood samples were collected from the hearts. After the liver was fixed with 4% paraformaldehyde, the tissue was sectioned and stained with hematoxylin–eosin (HE), Oil Red O (RUIBIO, Y07512), and Masson (Servicebio, G1006). Oil-red O staining is used to stain lipid droplets, and Masson staining is used to degrade collagen. Image Pro Plus software was used to quantitatively analyze the percentage of Oil Red O positive area to total area and the percentage of Masson positive area to total area.

Lkhagva et al. (2021) reported that the microbial composition of the colon and feces was very similar (Suzuki and Nachman, 2016), and the uncertainty of mouse excretion at the time of sampling. We selected colonic contents (formed fecal balls) 1 cm to 1.5 cm from the anus instead of feces. In addition, jejunal contents were 15 cm to 20 cm from the initial pylorus, ileum contents 1 cm to 5 cm from ileocecal valve, and cecal contents below the ileocecal valve selected as jejunum, ileum, and cecum samples, respectively. The contents of the jejunum, ileum, cecum, and colon were placed in liquid nitrogen and tested for the microbiome. The Animal Ethics Committee approved the experimental protocol of Southwest Medical University (No. of *Animal Ethics* Approval: SWMU2019463).

#### Biochemical analysis of serum

After anesthetization, blood samples were collected in the morning through cardiac puncture, and were centrifuged at 3500 rpm for 10 min at 4°C. Recovered supernatants were separated into 200 μl tubes and frozen at −80°C. Biochemical indices such as Alanine aminotransferase (ALT), Aspartate aminotransferase (AST), triglyceride (TG), total cholesterol (TC), high-density lipoprotein (HDL), and low-density lipoprotein (LDL) were detected by a fully automatic veterinary biochemical analyzer (Jiangxi Tekang Technology Co., Ltd., TC220).

### Microbiota analyses

DNA from the contents of the jejunum, ileum, cecum, and colon were isolated using the Qiagen Gel Extraction Kit (Qiagen, Hilden, Germany). The genomic DNA was amplified using fusion primers (341F, CCTACGGGRBGCASCAG; 806R: GGACTACNNGGGTATCTAAT) targeting the 16S V3-V4 rRNA gene with indexing barcodes. TruSeq^®^ DNA PCR-Free Sample Preparation Kit was used to establish the DNA library, and Qubit and Q-PCR quantified the library. All samples were pooled for sequencing on the Illumina HiSeq^™^ 2000 Sequencing system according to the manufacturer’s specifications.

Raw sequence tags were generated from FLASH (V1.2.7; *FLASH*, 2019^1^; Magoc and Salzberg, 2011). Quality filtering on the basic tags was performed under specific filtering conditions to obtain the high-quality clean labels according to the QIIME (V1.9.1; *Qiime*, 2019^2^; Caporaso et al., 2010) quality-controlled process. Chimera removal, the tags were compared with the reference database (Gold database) using the UCHIME algorithm (*UCHIME*, 2019)^3^ to detect chimera sequences. Then the chimera sequences were removed, and the Effective Tags were finally obtained. De novo operational taxonomic units (OTUs) clustering was carried out using the Uparse (V7.0.1001; *Uparse*, 2019^4^; Edgar, 2013), which identifies highly accurate OTUs from amplicon sequencing data with an identity threshold of 97%. Then the OTUs were used to screen effective sequences using Mothur (*Mothur*, 2019^5^; Schloss et al., 2009). The representative sequences of OTUs were used to analyze alpha-diversity (Chao1, Ace, Shannon and Simpson diversity index) based on their relative abundance. A heatmap was generated according to the relative abundance of OTUs by R software (V2.15.3; Gatto et al., 2015). Non-metric multidimensional scaling (NMDS) and Principal Coordinates analysis (PCoA) based on UniFrac distance was performed with Qiime (V1.9.1; *Qiime*, 2019 (see footnote 2); Caporaso et al., 2010). The linear discriminant analysis (LDA) with effect size measurements (LEfSe) was used to identify bacterial indicator groups specialized within the two groups, and species with LDA scores > 4 were considered biological markers. PICRUSt2 (*Picrust*, 2022^6^; Douglas et al., 2020) was used to predict the metabolic pathways of intestinal microbiota and investigate the functional differences in the microbial communities in samples from the four regions. OTUs with an alignment ratio lower than 0.8 were excluded (Langille et al., 2013; Liu et al., 2021).

### Statistical analysis

Data of body weight, serum indexes (TC, TG, HDL, LDL, AST, and ALT), and relative abundance of gut microbiota genes in metabolic pathways are presented as mean ± SD. Student’s *t*-test was used to determine the significance of the body weight, the relative amount of lipid droplets, the amount of collagen, and serum indexes (TC, TG, HDL, LDL, AST, and ALT). *Wilcox Rank-Sum test* was used to determine the significance of the alpha diversity. *Kruskal-Wallis test* was used to assess the importance of the relative abundance of major bacteria at phylum, family and genus levels and the relative abundance of primary and secondary functional genes in different intestinal segments within the same group. *Wilcox Rank-sum test* was used for the same intestinal segment between the two groups. A value of *p* < 0.05 was considered statistically significant. Statistical analysis was performed with SPSS software (Version 25, SPSS Inc., Chicago, United States).

## Results

### The animal model of NAFLD was successfully established

**Figure 1:**
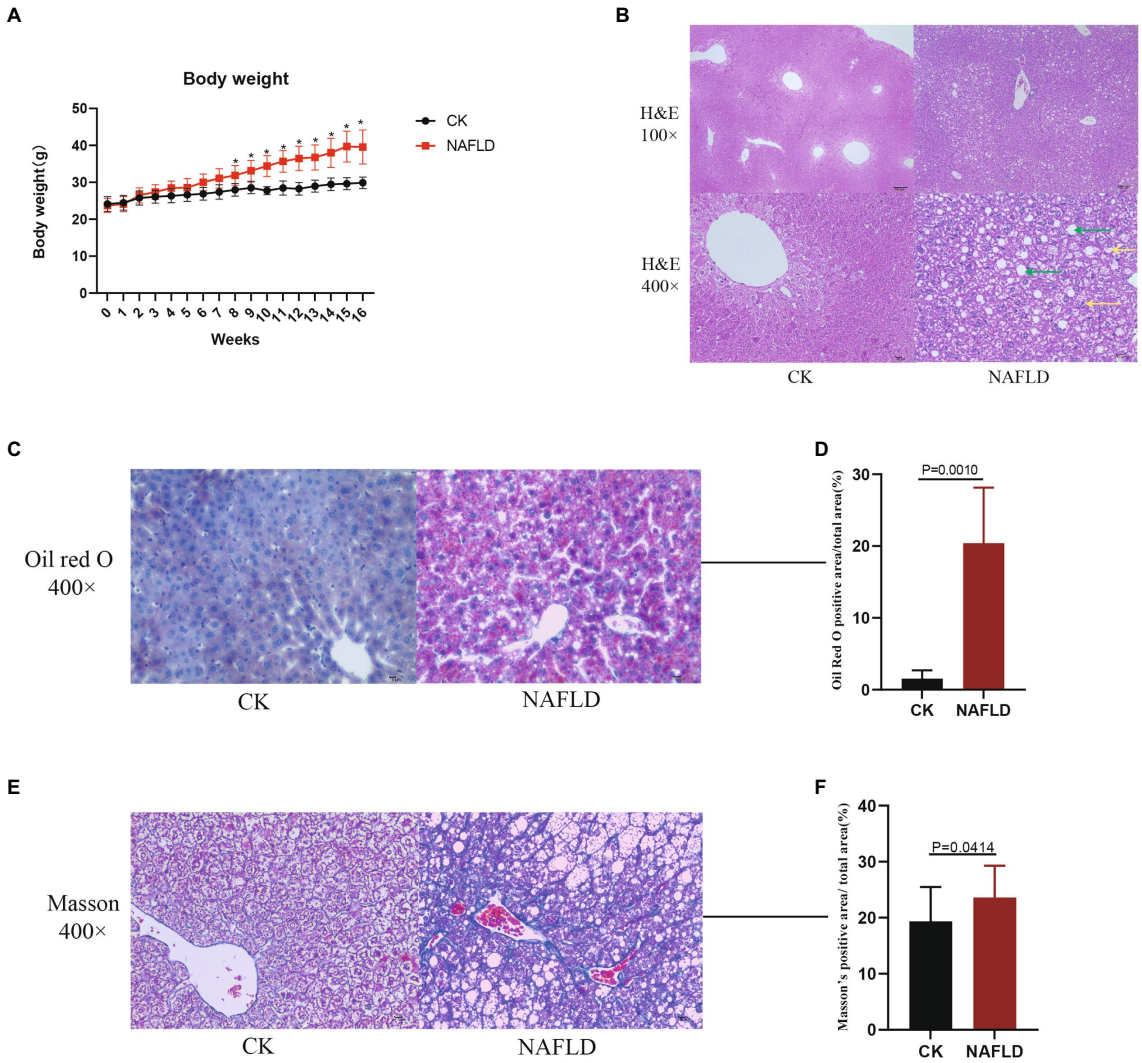
The body weight and Histopathology with Control and NAFLD mice. **(A)** Body weight changes in ND or HFD fed mice over 16 weeks. **(B)** Representative pictures of hematoxylin and eosin (H&E) staining for liver (100× and 400×), the green arrows indicate hepatic cell steatosis, and the yellow arrows indicate hepatic cell balloon-like changes. **(C,D)** Representative pictures of Oil red O staining for liver (400×) and quantitative analysis of lipid droplets relative amount. **(E,F)** Representative pictures of Masson staining for liver (400×) and quantitative analysis of the relative amount of collagen. Significance was assessed by Student *t-test*.

Twenty mice were randomly divided into CK and NAFLD groups. The CK group was fed a normal diet (ND), and the NAFLD group was fed a high-fat diet (HFD). After mice were fed HFD for 8 weeks, the body weights of NAFLD mice (31.9 ± 2.67 g) were markedly increased and significantly different from CK mice (27.97 ± 1.70 g; *p* = 0.031; Figure 1A). To produce obvious steatosis of the liver, we fed them the high-fat diet continuously for 16 weeks, and the body weight of NAFLD mice reached 39.58 ± 4.61 g, significantly higher than that of the CK group (29.90 ± 1.54 g; *p* = 0.033; Figure 1A).

Liver samples were collected for HE staining, and it was found that hepatic cell vacuolation and steatosis were observed in the liver tissues of NAFLD mice. Some hepatic cells showed obvious balloon-like changes, with different degrees of necrosis accompanied by inflammatory cell infiltration and cracks around the central vein. Pathological changes of hepatic cell necrosis were also observed around the cracks (Figure 1B). In the CK group, the liver cells were polygonal and arranged as hepatic cords, radially distributed around the central vein. There were large and round nuclei in the center of the cells, with uniform cytoplasm, no lipid droplets, and no steatosis or inflammatory cell infiltration (Figure 1B). By Oil Red O staining and calculating the relative amount of lipid droplets, it was found that the number of lipid droplets in the NAFLD group was significantly higher than that in the CK group (*p* = 0.0010; Figures 1C,D). Through Masson staining and the calculation of the relative amount of collagen, it was found that the fibrosis degree of NAFLD mice was significantly higher than that of CK mice, indicating that a certain degree of fibrosis had occurred in the liver of NAFLD mice (*p* = 0.0414; Figures 1E,F).

**Figure 2:**
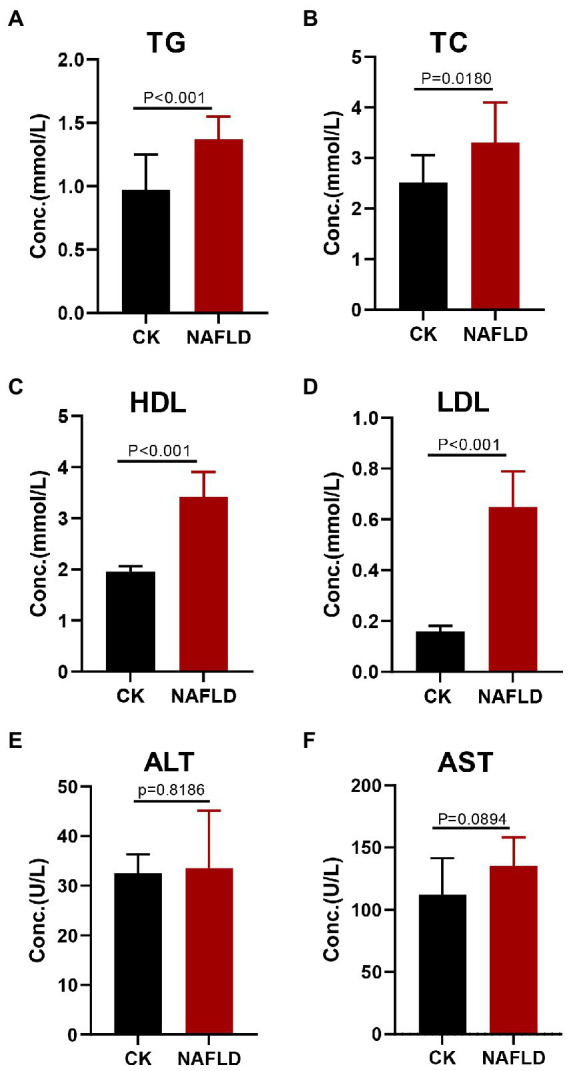
Serum lipid profiles and liver function indicators in Control and NAFLD mice. **(A)** Triglycerides (TG), **(B)** Total cholesterol (TC), **(C)** high density lipoprotein (HDL), **(D)** low density lipoprotein (LDL), **(E)** alanine aminotransferase (ALT), and **(F)** aspartate aminotransferase (AST). Significance was assessed by Student *t-test*.

Serological tests found that the NAFLD mice exhibited increased levels of triglyceride (TG), total cholesterol (TC), high-density lipoprotein (HDL), and low-density lipoprotein (LDL) in the serum (*p* < 0.05; Figures 2A–D), but Alanine aminotransferase (ALT, *p* = 0.8186) and Aspartate aminotransferase (AST, *p* = 0.0894) levels did not change significantly (Figures 2E,F).

The significant increase in body weight of mice in the HFD group, the discovery of hepatocyte steatosis, the rise in the number of lipid droplets, the aggravation of liver tissue fibrosis and the significant increase in serum triglycerides indicated that the HFD diet was successfully used to induce NAFLD model in mice (Lau et al., 2017).

### Alpha diversity of intestinal microbiota in CK and NAFLD mice

**Figure 3:**
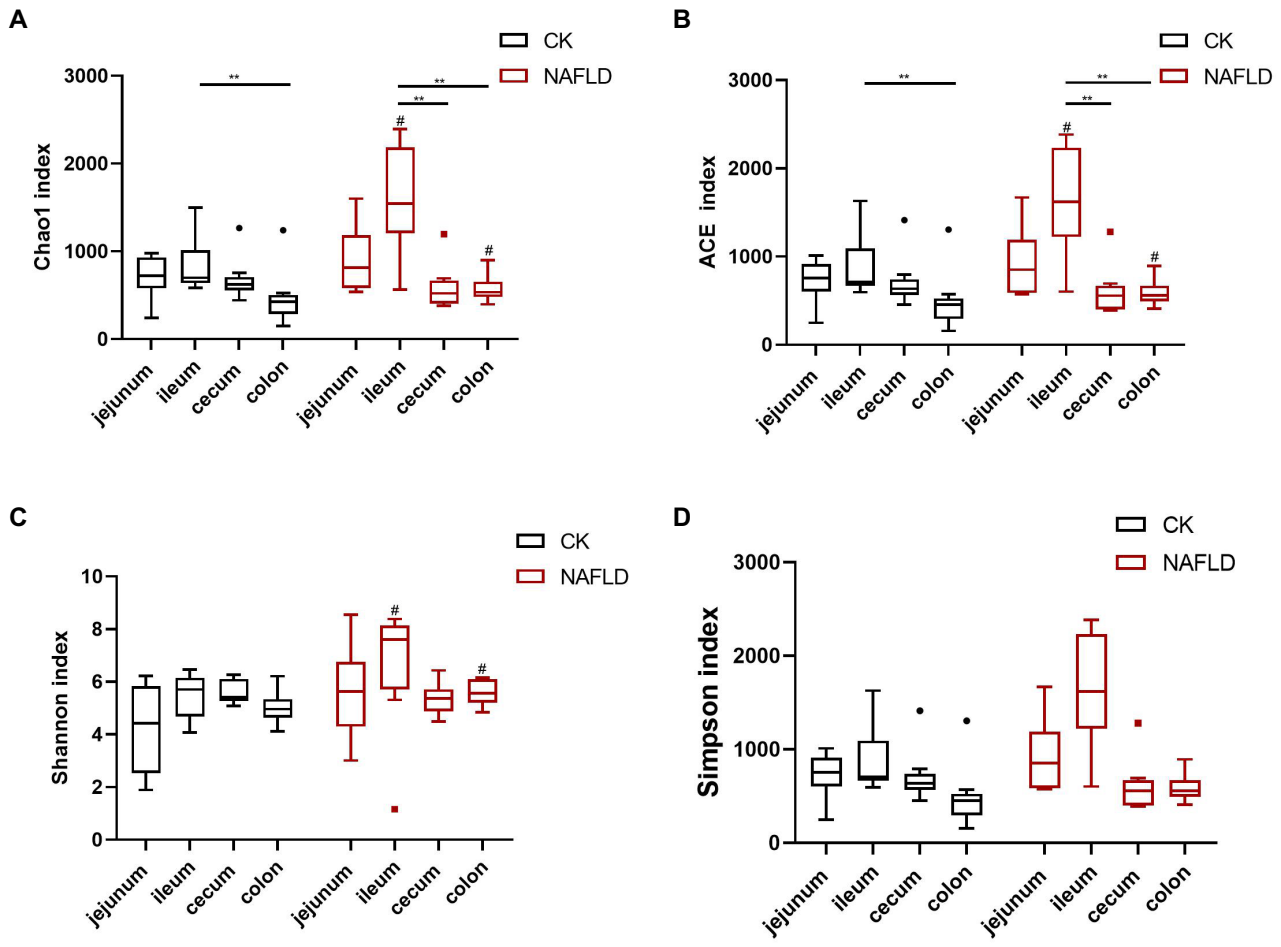
Alpha diversity analysis of the bacterial community in the jejunum, ileum, cecum, and colon of Control and NAFLD mice. **(A)** Chao1 index, **(B)** ACE index, **(C)** Shannon index, and **(D)** Simpson index. Significance was assessed by *Wilcox Rank-Sum test*. *indicate significant difference in the different intestinal segments in the CK group and NAFLD group. ^#^indicates the significant difference in the same intestinal segments between CK group and NAFLD group. *or^#^ *p* < 0.05; **or^##^ *p* < 0.01.

We used Chao1, ACE, Shannon and Simpson indices to evaluate the alpha diversity of various intestinal sections in normal and NAFLD mice. Microbial diversity was analyzed using the Shannon and Simpson indices, and richness was examined using Chao1 and ACE indices (Figure 3). In CK mice, the Chao1 and ACE indices values in the ileum were significantly higher than in the colon (*p* < 0.01; Figures 3A,B). In NAFLD mice, Chao1 and ACE indices in the ileum also yielded significantly higher values than those in the cecum and colon (*p* < 0.01). The Shannon index and Simpson index values in the jejunum, ileum, cecum, and colon had no significant differences (*p* > 0.05; Figures 3C,D). Compared with CK mice, the Simpson index value of NAFLD mice did not change significantly in the four intestinal segments (*p* > 0.05; Figure 3D). Still, in the ileum and colon, the Chao1, ACE and Shannon index values of NAFLD mice changed significantly (*p* < 0.05; Figures 3A–C). These results indicate that the ileum of CK mice has the highest microbiota diversity, which is substantially higher than that of the colon. The ileum of NAFLD mice has a significantly higher microbiota diversity than the cecum and colon. Compared with CK mice, while the diversity of microbiota in the jejunum and cecum of NAFLD mice did not change significantly, it was considerably greater in the ileum and colon.

### Beta diversity of intestinal microbiota in NAFLD mice was changed

**Figure 4:**
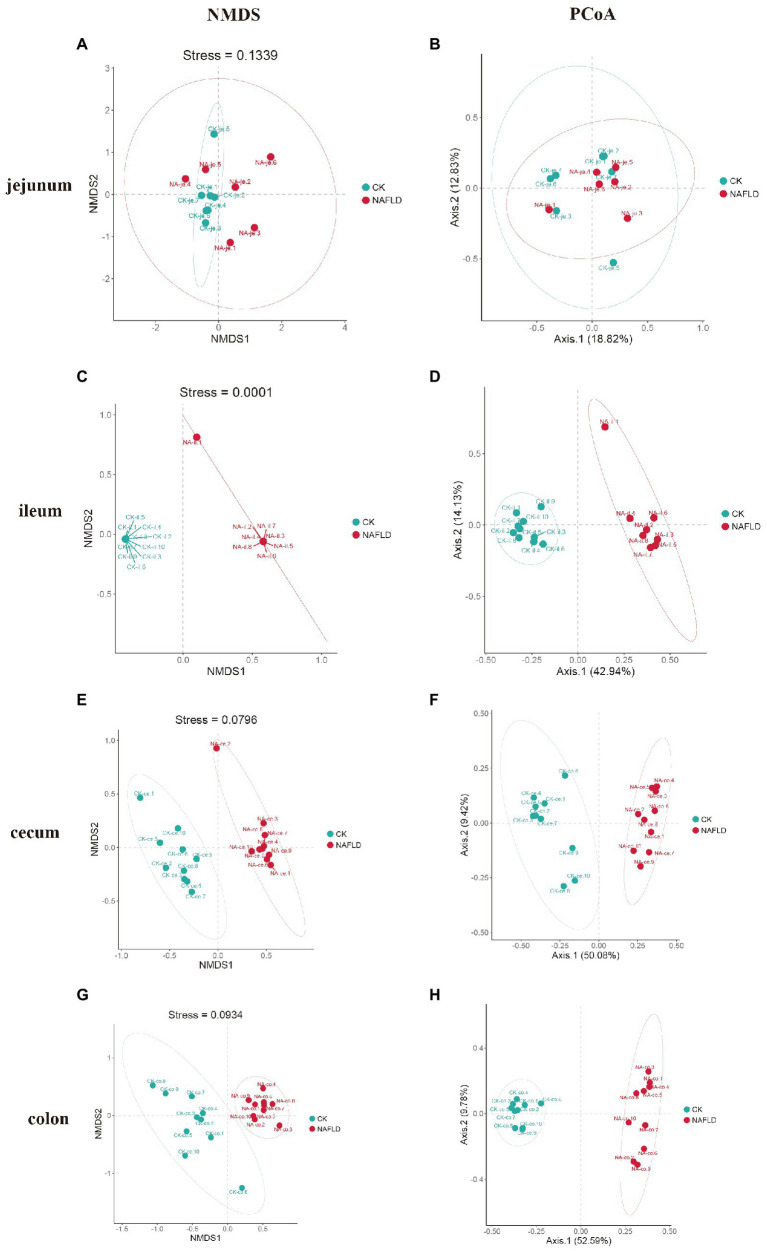
Beta diversity analysis of the same intestinal segments between CK group and NAFLD group assessed by NMDS and PCoA based on Bray-Curtis distance. **(A,B)** Jejunum, **(C,D)** ileum, **(E,F)** cecum, and **(G,H)** colon.

Non-metric Multidimensional Scales (NMDS) based on Bray-Curtis distances and Principal Co-ordinate Analysis (PCoA) based on Weighted and Unweighted Unifrac distances were carried out to reveal the differences in the nonlinear and linear bacterial community structure of the samples, respectively. The NMDS and PCoA showed that the distribution regions of jejunum samples from NAFLD mice overlapped with those from CK mice in the two-dimensional coordinate system (Figures 4A,B). However, the distribution regions of the ileum (Figures 4C,D), cecum (Figures 4E,F), and colon (Figures 4G,H) samples from NAFLD mice were separated from those of CK mice.

**Figure 5:**
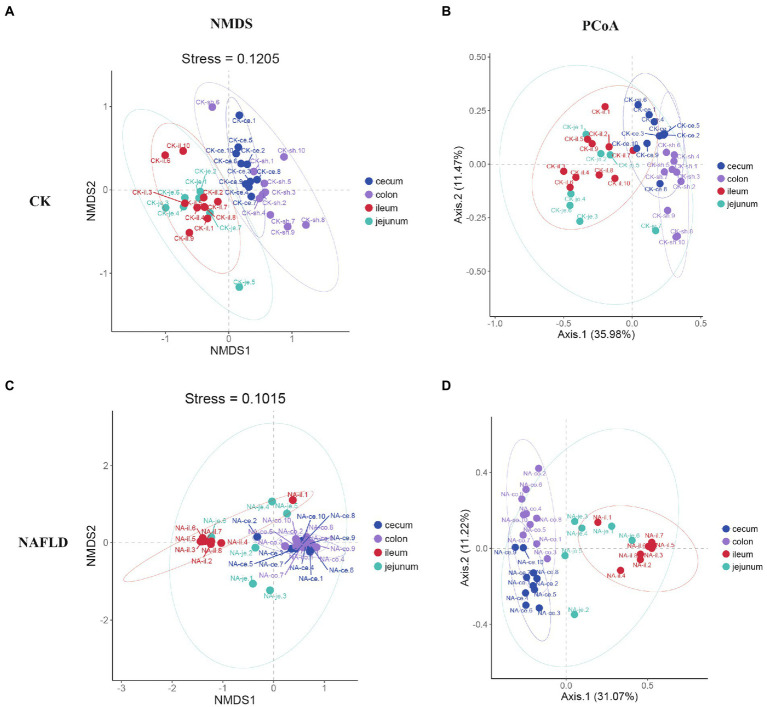
Beta diversity analysis of the jejunum, ileum, cecum, and colon in the same group assessed by NMDS and PCoA based on Bray-Curtis distance. **(A,B)** CK group, and **(C,D)** NAFLD group.

Although PCoA analysis found that the distribution regions of samples from four different intestinal segments overlapped in both CK mice and NAFLD mice, NMDS analysis showed that the large intestine (cecum and colon) and small intestine (jejunum and ileum) samples from CK mice were shown to be distinct (Figures 5A,B). However, in NAFLD mice, there was significant overlap in the distribution regions of all four intestinal segments. This indicates that following a high-fat diet, the intestinal microbiota structure of the ileum, cecum, and colon of NAFLD mice changes, resulting in the microbiota structure of the large intestine and small intestine, which should be different, sharing more similarities (Figures 5C,D).

### Composition of the intestinal microbiota of CK and NAFLD mice

#### The observed number of OTUs in the guts of CK and NAFLD mice

**Figure 6:**
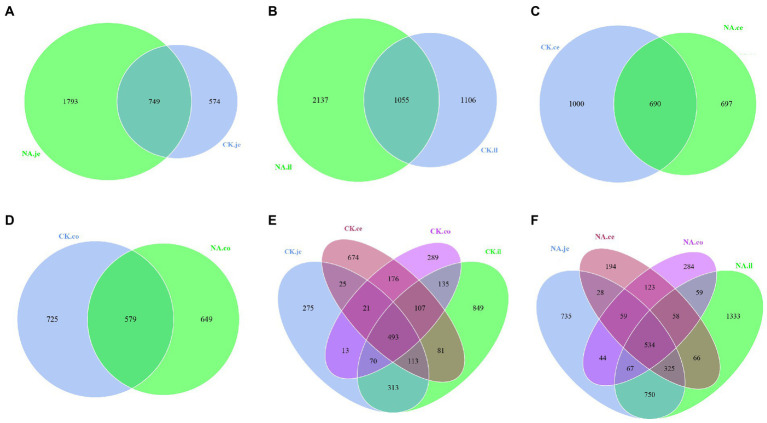
Venn diagram based on OTU. **(A)** The jejunum between CK and NAFLD (NA) groups, **(B)** the ileum between CK and NAFLD (NA) groups, **(C)** the cecum between CK and NAFLD (NA) groups, **(D)** the colon between CK and NAFLD (NA) groups, **(E)** the jejunum, ileum, cecum, and colon of CK group, and **(F)** the jejunum, ileum, cecum, and colon of NAFLD (NA) group.

Venn graph is used to show the distribution and differences of OTU in four intestinal segments of NAFLD mice and CK mice (Figure 6). The Venn graph showed that in jejunum and ileum, the number of OTUs in the NAFLD group was 1,219 and 1,031 more than in the CK group (Figures 6A,B). In the cecum and colon, the number of OTUs in the NAFLD group was 303 and 76 less than in the CK group (Figures 6C,D). In CK mice and NAFLD mice, the number of OTUs in the ileum was the highest, followed by jejunum and cecum, and the colon was the least (Figures 6E,F). This indicates that after a high-fat diet, the richness of small intestinal (jejunum and ileum) microbiota in NAFLD mice increases, while the richness of the large intestine (cecum and colon) decreases. The richness of the small intestine is more than that of the large intestine, and the richness of ileal microbiota is the most abundant. The jejunum and ileum microbiota had the highest number of common OTUs.

#### Relative abundance of major bacteria in the intestinal tract of CK and NAFLD mice

**Figure 7:**
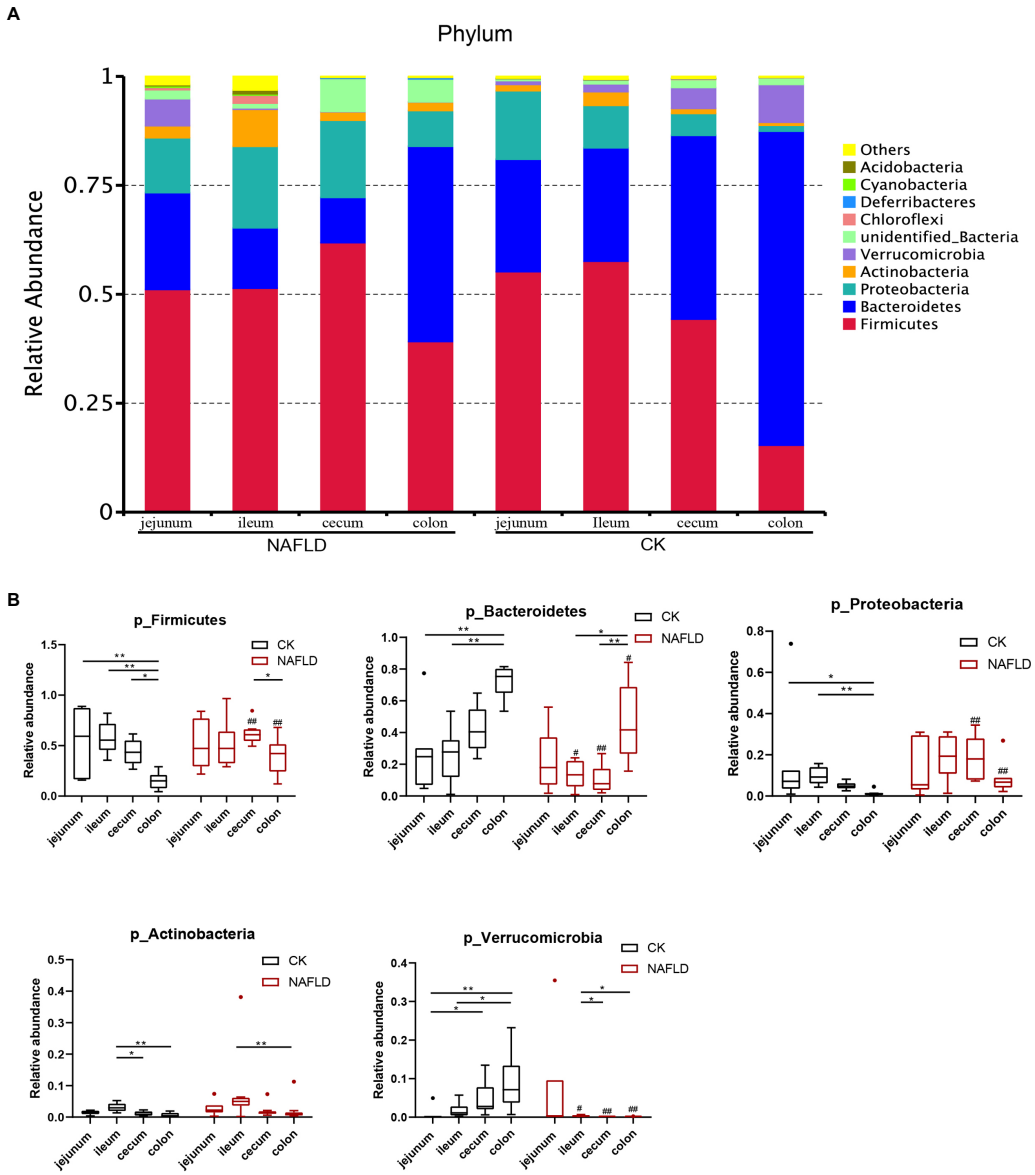
Relative abundance of bacteria at phylum level. **(A)** Stacked column graph of the top 10 phyla with the highest relative abundance. **(B)** The box diagram was used to analyze the differences in relative abundance of the five phyla with the highest relative abundance. *Wilcox Rank-sum* test was used to compare the significance of the same intestinal segment between the CK group and the NAFLD group; *Kruskal-wallis test* was used to compare the differences between different intestinal segments in the same group. *indicate significant difference in the different intestinal segments in the CK group and NAFLD group. ^#^indicates the significant difference in the same intestinal segments between CK group and NAFLD group. *or^#^ *p* < 0.05; **or^##^ *p* < 0.01.

We screened the 10 phyla with the highest abundance and displayed the results in a stacked column chart (Figure 7A; Supplementary Table S2). We selected five phyla with the highest abundance using the *Rank Sum test* (Figure 7B; Supplementary Table S2), which accounted for more than 90% of the community abundance at the phylum level. The dominant phyla were *Firmicutes*, *Bacteroidetes*, *Proteobacteria*, *Actinobacteria* and *Verrucomicroibia*. *Firmicutes* were the highest in the jejunum, ileum, and cecum, and *Baceteroidetes* was the highest in the colon. In the gut of CK mice, none of these five phyla differed relative abundance between jejunum and ileum, and only *Fimictutes* differed relative abundance between ileum and cecum (*p* < 0.05). However, there are many differences in the relative abundance of microbiota at the phylum level between the small intestine and the large intestine. The relative abundance of *Firmicutes*, *Bacteroidetes*, *Proteobacteria* and *Verrucomicrobia* in the jejunum and ileum is significantly different from that in the colon (*p* < 0.05).

In addition, the relative abundance of *Verrucomicrobia* in the jejunum was significantly lower than that in the ileum (*p* < 0.05). The relative abundance of *Actinobacteria* in the ileum was substantially higher than that in the cecum and colon (*p* < 0.05). But after the high-fat diet, the differences in the relative abundance of the five phyla became smaller among the four segments. In the intestine of NAFLD mice, *Proteobacteria* showed no difference in relative abundance among four intestinal segments. There was also no difference in the relative abundance of *Firmicutes* between the small and large intestines. Compared with the CK group, after a high-fat diet, there was no significant change in the jejunal microbiota of NAFLD mice. Still, *Firmicutes* significantly decreased, and *Verrucomicrobia* increased dramatically in the ileum (*p* < 0.05). *Firmicutes* and *Proteobacteria* were significantly increased in the cecum and colon (*p* < 0.05), while *Bacteroidetes* and *Verrucomicrobia* were considerably reduced (*p* < 0.05). At the family level, we found the same pattern. After the high-fat diet, the differences in the relative abundance of major families among the four segments became smaller. Compared with CK mice, the relative abundance of bacteria in the jejunum of NAFLD mice was significantly changed in a few cases, only one family. More bacteria in the ileum, cecum and colon had significant changes at the family level. But the family-level bacteria that changed in the cecum and colon and the trends were more similar (Supplementary Figures S1A,B; Supplementary Table S3).

**Figure 8:**
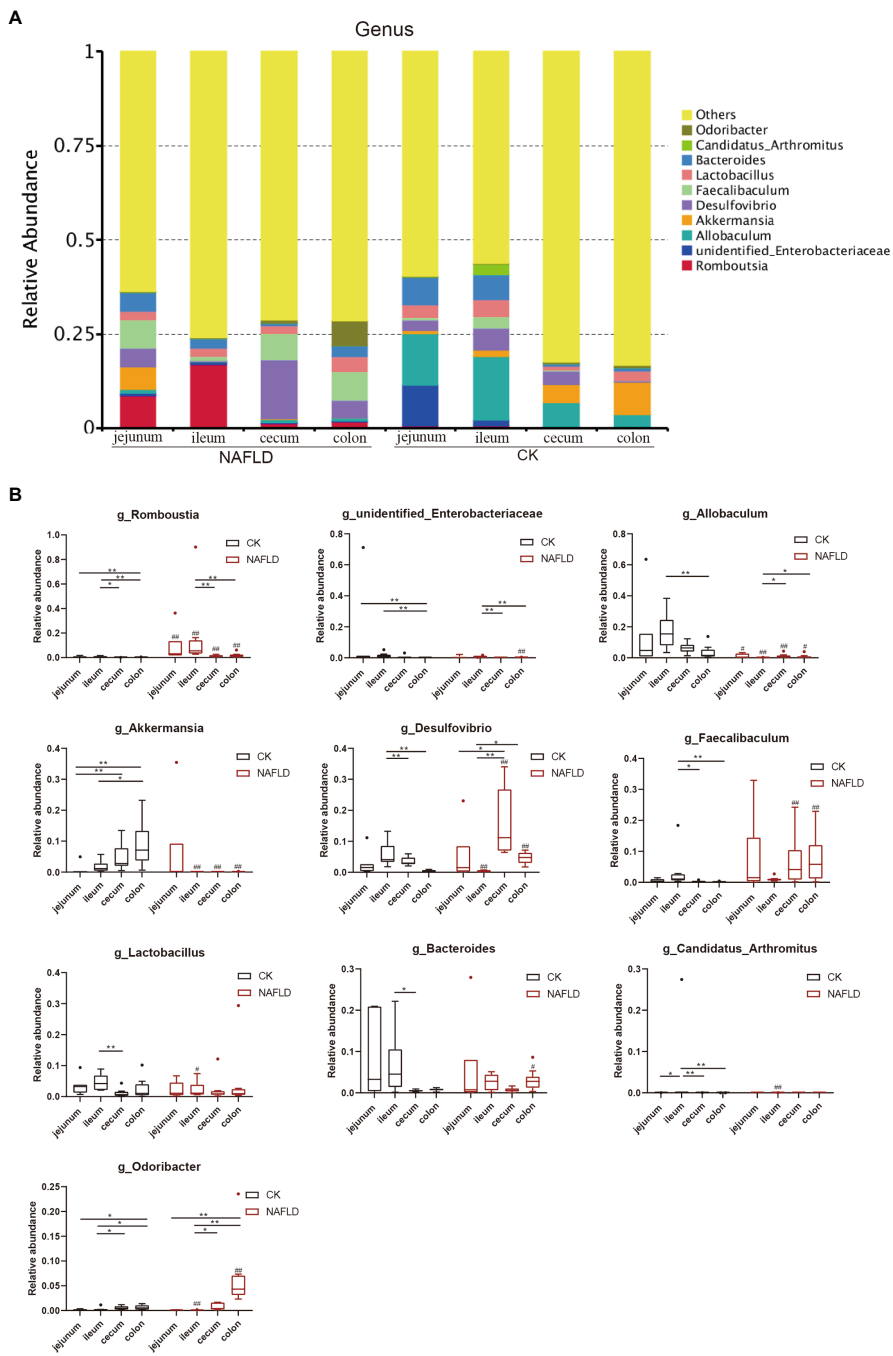
Relative abundance of bacteria at genus level. **(A)** Stacked column graph of the top 10 genera with the highest relative abundance. **(B)** The box diagram was used to analyze the differences in relative abundance of the five genera with the highest relative abundance. *Wilcox Rank-sum test* was used to compare the significance of the same intestinal segment between the CK group and the NAFLD group; *Kruskal-wallis test* was used to compare the differences between different intestinal segments in the same group. *indicate significant difference in the different intestinal segments in the CK group and NAFLD group. ^#^indicates the significant difference in the same intestinal segments between CK group and NAFLD group. *or^#^ *p* < 0.05; **or^##^ *p* < 0.01.

At the genus level, we selected the 10 genera with the highest abundance for the stacked column chart (Figure 8A; Supplementary Table S4), and the relative abundance of these 10 genera accounted for 17–36%. The top 10 genera with the highest relative abundances were plotted in boxplots, and the *Rank Sum test* was performed (Figure 8B; Supplementary Table S4). The results showed that the relative abundance of *Candidatus_Arhromitus* was significantly different between jejunum and ileum in CK mice (*p* < 0.05). There were no significant differences in the relative abundance of genera between the cecum and colon. There is a large difference between the small and large intestine, *Romboutsia* was significantly different between jejunum and ileum (*p* < 0.05), and *Romboutsia*, *unidentified Enterobacteriaceae*, *Akkermansia* and *Odoribacter* were different between jejunum and colon (*p* < 0.05). There were significant differences between ileum and cecum in seven genera, including *Romboutsia*, *Desulfovibrio*, *Faecallbaculum*, *Lactobacillus*, *Bacteroides*, *Candidatus Arhromitus*, and *Odoribacter* (*p* < 0.05). There were substantial differences between ileum and colon in eight genera, including *Romboutsia*, *unidentified Enterobacteriaceae*, *Allobaculum*, *Akkermansia*, **Desul*f*ovibrio**, *Faecallbaculum*, *Candidatus Arhromitus*, and *Odoribacter* (*p* < 0.05). After the high-fat diet, the differences in microbial abundance between intestinal segments were reduced. The *Romboutsia* has become no significant difference in the jejunum and cecum of NAFLD mice, and *Desulfovibrio* is still significantly different (*p* < 0.05); only *Odoribacter* was significantly different between the jejunum and colon (*p* < 0.05); only five genera, such as *Romboutsia*, *unidentified Enterobacteriaceae*, *Akkermansia*, *Desulfovibrio* and *Odoribacter* showed significant differences between ileum and cecum (*p* < 0.05); Five genera, *Romboutsia*, *unidentified Enterobacteriaceae*, *Allobaculum*, *Desulfovibrio*, and *Odoribacter*, were significantly different between the ileum and colon (*p* < 0.05). Compared with the CK group, only two genera were changed in the jejunum of NAFLD mice, and the relative abundance of *Romboutsia* was significantly increased, and *Allobaculum* was decreased considerably (*p* < 0.05); seven genera changed in the ileum, the relative abundance of *Romboutsia and Candidatus Arhromitus* was improved greatly, while *Allobaculum*, *Akkermansia*, *Desulfovibrio*, *Lactobacillus* and *Odoribacter* were significantly decreased (*p* < 0.05). The relative abundance of 5 genera changed substantially in the cecum, and these 5 genera also changed significantly in the colon (*p* < 0.05). The changing trend was consistent: *Romboutsia* and *Akkermansia* were significantly decreased, while *Allobaculum*, *Desulfovibrio* and *Faecallbaculum* were significantly increased. In addition, three genera in the colon showed significant changes.

These results indicate that the major bacterial compositions of the small intestine (jejunum and ileum) and large intestine (cecum and colon) were quite different. After a high-fat diet, the jejunum’s major bacterial compositions had fewer changes. In contrast, the ileum, cecum, and colon had greater changes, and the characteristics of the changes to the major bacterial arrangements of the cecum and colon were closer.

#### Bacterial taxonomic biomarkers in the intestinal tract of CK and NAFLD mice

**Figure 9:**
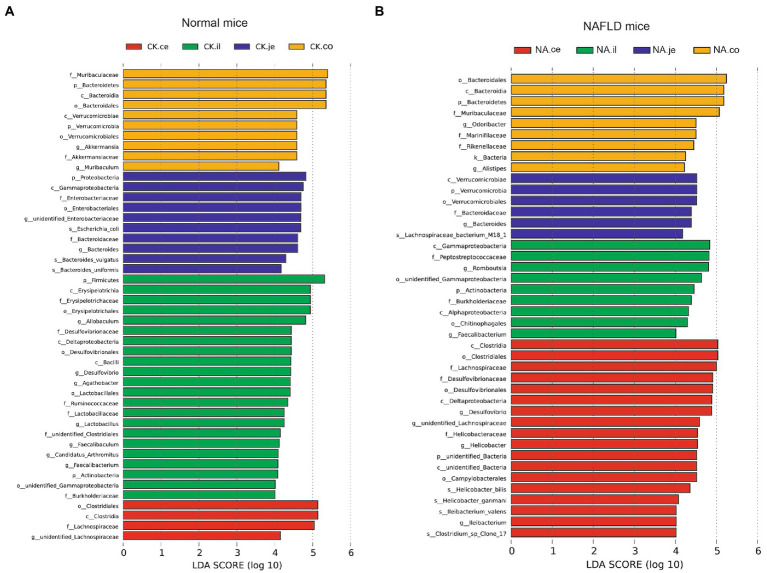
Linear discriminant analysis effect size (LEfSe) analysis of microbiota among different intestinal segments in the same group. **(A)** CK group, and **(B)** NAFLD (NA) group (*α* = 0.05, logarithmic Linear Discriminant Analysis (LDA) score threshold = 4.0).

**Figure 10:**
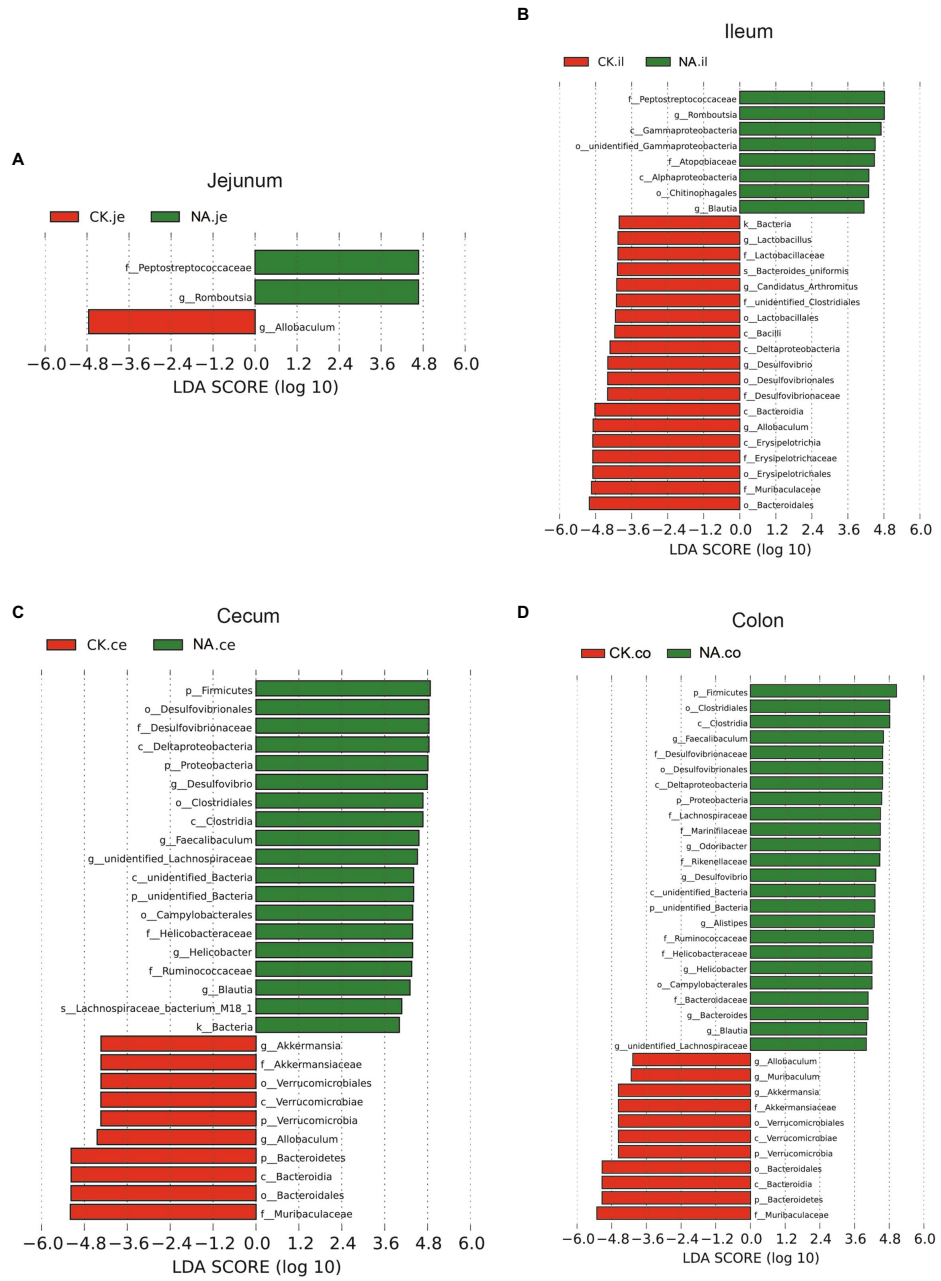
LEfSe analysis of intestinal microbiota in the same segment between CK group and NAFLD (NA) group. **(A)** Jejunum; **(B)** Ileum; **(C)** Cecum; **(D)** Colon (*α* = 0.05, logarithmic Linear Discriminant Analysis (LDA) score threshold = 4.0).

Linear discriminant analysis Effect Size (LEfSe) was used to examine the relative abundance of different bacteria taxa (from phylum to genus) in samples from another region (Figures 9, 10). Species with LDA scores >4 were considered biological markers. A comparison between the four different intestinal segments in CK mice found only 4 biomarkers in the cecum, up to 22 in the ileum, and 10 in the jejunum and colon (Figure 9A). When comparing the four different intestinal segments in NAFLD mice, the maximum number of biomarkers was 18 in the cecum, 9 in the ileum and colon, and the lowest number was 6 in the jejunum (Figure 9B).

While the biomarker of CK mice in the jejunum was *Allobaculum*, those of NAFLD mice were *Peptostreptococcaceae* and *Romboustsia* (Figure 10A). In the ileum, 19 biomarkers were found in CK mice and 8 in NAFLD mice (Figure 10B). In the cecum, there were 10 biomarkers in CK mice and 19 biomarkers in NAFLD mice (Figure 10C). In the colon, 11 biomarkers were found in CK mice and 24 in NAFLD mice (Figure 10D). Compared to CK mice, NAFLD mice had more common biomarkers in the cecum and colon and fewer in the jejunum and ileum. In CK mice, at the genus level, there was one common biomarker, *Allobaculum*, in all four intestinal segments and two in the cecum and colon. In NAFLD mice, at the genus level, five biomarkers in the cecum of NAFLD mice, *Blautia*, *Desulfovibrio*, *Faecalibaculum*, *Helicobacter*, and *unidentified Lachnospiraceae*, were also the biomarkers in the colon. Interestingly, in a previous study, *Blautia*, *Faecalibaculum*, *Helicobacter* and *unidentified Lachnospiraceae* are significantly increased in NAFLD mice (Gu et al., 2022). However, the ileum and large intestine (cecum and colon) shared only a few biomarkers, with only one common biomarker at the genus level.

### Predicted function of intestinal microbiota

Based on the 16S rDNA sequence, PICRUSt2 was used to predict the function of intestinal flora, and the relative abundance of metabolic function genes was the highest in the primary function, accounting for about 70%. In the CK group, the colon had a significantly higher relative abundance of metabolic function genes than other intestinal segments but had a lower relative abundance of other primary function genes (*p* < 0.05, Table 1). In NAFLD mice, except for human diseases, other functional genes enriched in the colon were not significantly different from those in the jejunum and ileum (*p* > 0.05, Table 1). Compared with the CK group, there was no significant difference in other metabolic pathways in the jejunum and ileum of NAFLD mice (*p* > 0.05), except that the abundance of genes related to cellular processes in the ileum was significantly increased (*p* < 0.05, Table 1). In the cecum and colon, the abundance of genes related to metabolism was significantly lower in NAFLD mice (*p* < 0.05), along with the abundance of genes related to organismal systems in the cecum (*p* < 0.05, Table 1). However, the abundance of genes related to genetic information processing, cellular processes, and environmental information processing was significantly higher in the cecum and colon of NAFLD mice (*p* < 0.05, Table 1).

**Table 1:** Relative abundance of primary functional genes in gut microbiota of normal and NAFLD mice.

Functions	CK-JE	CK-IL	CK-CE	CK-CO	NA-JE	NA-IL	NA-CE	NA-CO
Metabolism	71.87 ± 2.03^b^	71.72 ± 1.66^b^	72.97 ± 1.16^b^	75.78 ± 0.71^a^	71.76 ± 2.10^AB^	71.20 ± 1.31^AB^	69.70 ± 0.69^A*^	72.59 ± 1.87^B*^
Genetic Information	12.02 ± 1.52^ab^	12.19 ± 0.65^a^	11.49 ± 0.27^bc^	10.98 ± 0.19^c^	11.76 ± 0.86	11.53 ± 0.78	12.14 ± 0.50^*^	11.79 ± 0.80^*^
Processing								
Cellular Processes	5.51 ± 1.04^a^	5.66 ± 0.80^a^	5.85 ± 0.82^a^	4.16 ± 0.49^b^	6.16 ± 1.25^A^	6.65 ± 0.70^A*^	7.80 ± 0.81^B*^	5.77 ± 1.14^A*^
Human Diseases	5.49 ± 0.9^a^	5.28 ± 0.24^a^	5.02 ± 0.11^ab^	4.82 ± 0.07^b^	5.30 ± 0.29^AB^	5.46 ± 0.19^A^	5.11 ± 0.23^B^	5.01 ± 0.20^C^
Environmental Information Processing	2.77 ± 0.8^ac^	2.81 ± 0.35^a^	2.38 ± 0.23^c^	1.83 ± 0.11^b^	2.63 ± 0.42^AB^	2.76 ± 0.26^AB^	3.10 ± 0.27^A*^	2.44 ± 0.40^B*^
Organismal Systems	2.34 ± 0.09^ab^	2.34 ± 0.08^a^	2.28 ± 0.06^a^	2.43 ± 0.02^b^	2.38 ± 0.19^A^	2.41 ± 0.13^A^	2.16 ± 0.09^B*^	2.4 ± 0.11^A^

1. Lowercase letters, such as a, b, c, are used to indicate significant difference in the relative abundance of functional genes of the microbiota among different intestinal segments in the CK group. Capital letters, such as A, B, C, are used to indicate significant difference in the relative abundance of functional genes of the microbiota among different intestinal segments in NAFLD (NA) group. The presence of identical letters in the superscript of the relative abundance of functional genes indicates no significant differences between groups (*p* > 0.05), while the absence of identical letters indicates significant differences between groups (*p* < 0.05). 2. “*” and “**” indicates the significant difference in the relative abundance of functional genes in the same intestinal microbiota between CK group and NAFLD (NA) group. *p < 0.05; **p < 0.01.

Among the secondary functional pathways, amino acid metabolism and carbohydrate metabolism were the two secondary metabolic pathways with the highest relative abundance, followed by cofactor and vitamin metabolism. In the CK group, the colon had the lowest relative abundance of amino acid metabolism and carbohydrate metabolism function (*p* < 0.05) and a significantly higher relative abundance of cofactors and vitamin metabolism function than the ileum and cecum (*p* < 0.05, Table 2). However, there was no difference in these functions between the jejunum, ileum, and colon in the NAFLD group (*p* > 0.05), although significant differences were observed in the relative abundance of amino acid metabolism, cofactors and vitamin metabolism between the cecum and the other three intestine segments (*p* < 0.05, Table 2). Compared to CK mice, NAFLD mice had significantly higher levels of amino acid metabolism and carbohydrate metabolism in the cecum and colon (*p* < 0.05), a significantly lower relative abundance of carbohydrate metabolism in the ileum (*p* < 0.05), and significantly higher relative abundance of cofactors and vitamin metabolism in the cecum (*p* < 0.05, Table 2). Changes in the cecum and colon were similar in terms of the relative abundance of functional genes in the microbiota.

**Table 2:** Relative abundance of secondary functional genes in gut microbiota of normal and NAFLD mice.

Functions	CK-JE	CK-IL	CK-CE	CK-CO	NA-JE	NA-IL	NA-CE	NA-CO
Metabolism								
Amino acid metabolism	10.83 ± 1.02^ab^	10.95 ± 0.45^a^	10.96 ± 0.23^a^	10.48 ± 0.12^b^	10.90 ± 0.33^A^	10.96 ± 0.42^A^	11.35 ± 0.22^B*^	10.89 ± 0.48^A*^
Biosynthesis of other secondary metabolites	6.14 ± 0.64^a^	6.29 ± 0.24^ab^	6.54 ± 0.13^bc^	6.75 ± 0.10^c^	6.04 ± 0.35^AB^	5.75 ± 0.59^B*^	6.19 ± 0.15^AB*^	6.48 ± 0.34^A*^
Carbohydrate metabolism	10.96 ± 0.74^ab^	11.02 ± 0.30^a^	10.58 ± 0.25^b^	10.07 ± 0.13^c^	10.54 ± 0.61^AB^	10.15 ± 0.72^A*^	10.96 ± 0.27^B*^	10.56 ± 0.37^AB*^
Chemical structure transformation maps	0.09 ± 0.07^a^	0.12 ± 0.09^a^	0.23 ± 0.15^ab^	0.37 ± 0.25^b^	0.52 ± 0.67^B^	0.78 ± 0.46^B*^	0.02 ± 0.03^A*^	0.02 ± 0.03^A*^
Energy metabolism	4.53 ± 0.14^a^	4.55 ± 0.14^a^	4.39 ± 0.06^ab^	4.25 ± 0.07^b^	4.50 ± 0.20^B^	4.58 ± 0.13^AB^	4.73 ± 0.15^A*^	4.54 ± 0.16^B*^
Global and overview maps	5.78 ± 0.45^a^	5.8 ± 0.15^a^	5.68 ± 0.17^a^	5.33 ± 0.08^b^	5.77 ± 0.22^A^	5.82 ± 0.31^A^	6.05 ± 0.08^B*^	5.69 ± 0.23^A*^
Glycan biosynthesis and metabolism	5.69 ± 2.42^a^	5.85 ± 1.24^a^	7.45 ± 1.25^b^	10.24 ± 0.80^c^	5.54 ± 2.48^AB^	3.41 ± 0.60^C*^	4.04 ± 0.80^BC*^	6.85 ± 1.96^A*^
Lipid metabolism	4.34 ± 0.34^ab^	4.32 ± 0.17^a^	4.54 ± 0.14^b^	4.78 ± 0.13^c^	4.36 ± 0.50^AB^	4.89 ± 0.40^C*^	4.07 ± 0.22^A*^	4.42 ± 0.23^B*^
Metabolism of cofactors and vitamins	9.48 ± 1.11^ab^	9.07 ± 0.65^a^	9.27 ± 0.26^a^	9.97 ± 0.21^b^	9.62 ± 0.72^A^	9.52 ± 0.47^A^	8.93 ± 0.46^B^	9.51 ± 0.56^A*^
Metabolism of other amino acids	7.03 ± 0.51^ab^	6.96 ± 0.29^ab^	6.81 ± 0.11^a^	7.07 ± 0.14^b^	7.06 ± 0.70^AB^	7.55 ± 1.11^B^	6.68 ± 0.21^A^	7.02 ± 0.44^AB^
Metabolism of terpenoids and polyketides	2.86 ± 0.37	2.79 ± 0.20	2.75 ± 0.07	2.73 ± 0.07	2.71 ± 0.27^AB^	2.88 ± 0.20^A^	2.61 ± 0.19^B^	2.71 ± 0.20^AB^
Nucleotide metabolism	1.64 ± 0.13^a^	1.63 ± 0.05^a^	1.57 ± 0.03^b^	1.53 ± 0.03^b^	1.59 ± 0.14	1.57 ± 0.15	1.61 ± 0.06	1.60 ± 0.09^*^
Xenobiotics biodegradation and metabolism	2.48 ± 0.81^ab^	2.37 ± 0.11^a^	2.22 ± 0.09^ab^	2.22 ± 0.09^b^	2.62 ± 0.64^A^	3.35 ± 0.48^B*^	2.46 ± 0.27^A*^	2.28 ± 0.15^A^
Genetic Information Processing								
Folding, sorting and degradation	3.00 ± 0.28^a^	2.92 ± 0.10^ab^	2.81 ± 0.08^bc^	2.68 ± 0.04^c^	2.95 ± 0.19^AB^	3.04 ± 0.16^A^	3.01 ± 0.08^AB*^	2.88 ± 0.17^B*^
Replication and repair	5.53 ± 0.80^a^	5.65 ± 0.37^a^	5.32 ± 0.12^ab^	5.07 ± 0.10^b^	5.39 ± 0.46	5.18 ± 0.38	5.53 ± 0.31	5.43 ± 0.40^*^
Transcription	0.32 ± 0.08^ab^	0.34 ± 0.04^b^	0.28 ± 0.02^ac^	0.26 ± 0.01^c^	0.31 ± 0.04	0.29 ± 0.03	0.32 ± 0.03^*^	0.30 ± 0.04^*^
Translation	3.17 ± 0.56^ab^	3.29 ± 0.22^a^	3.09 ± 0.08^ab^	2.98 ± 0.06^b^	3.13 ± 0.27^AB^	3.02 ± 0.24^A^	3.28 ± 0.16^B*^	3.18 ± 0.23^AB*^
Cellular Processes								
Cell growth and death	1.89 ± 0.31	1.91 ± 0.05	1.97 ± 0.05	2.02 ± 0.06	1.90 ± 0.15^A^	1.92 ± 0.14^A^	1.92 ± 0.03^A*^	2.03 ± 0.05^B^
Cell motility	1.86 ± 0.96^a^	2.12 ± 0.78^a^	2.26 ± 0.80^a^	0.6 ± 0.46^b^	2.59 ± 1.14^B^	2.97 ± 0.80^B*^	4.16 ± 0.72^A*^	2.17 ± 1.12^B*^
Cellular community – eukaryotes	0.00 ± 0.00	0.00 ± 0.00	0.00 ± 0.00	0.00 ± 0.00	0.00 ± 0.00	0.00 ± 0.00	0.00 ± 0.00	0.00 ± 0.00
Cellular community – prokaryotes	1.46 ± 0.49^a^	1.32 ± 0.06^a^	1.26 ± 0.10^a^	1.05 ± 0.05^b^	1.36 ± 0.17^C^	1.50 ± 0.04^A*^	1.49 ± 0.11^A*^	1.22 ± 0.11^B*^
Transport and catabolism	0.30 ± 0.11^a^	0.31 ± 0.06^a^	0.37 ± 0.05^a^	0.49 ± 0.03^b^	0.31 ± 0.12^AB^	0.26 ± 0.07^B^	0.23 ± 0.04^B*^	0.35 ± 0.09^A*^
Human Diseases								
Cancer: overview	0.61 ± 0.07	0.58 ± 0.03	0.56 ± 0.01	0.58 ± 0.01	0.58 ± 0.06^BC^	0.62 ± 0.05^C^	0.48 ± 0.04^A*^	0.55 ± 0.06^B^
Cancer: specific types	0.09 ± 0.05^b^	0.08 ± 0.01^b^	0.06 ± 0.01^ab^	0.06 ± 0.01^a^	0.09 ± 0.05^AB^	0.15 ± 0.05^B*^	0.07 ± 0.01^A^	0.07 ± 0.01^A*^
Cardiovascular disease	0.17 ± 0.02^a^	0.16 ± 0.01^ab^	0.16 ± 0.01^ab^	0.16 ± 0.01^b^	0.18 ± 0.03^AB*^	0.22 ± 0.02^B^	0.16 ± 0.00^A^	0.17 ± 0.01^A*^
Drug resistance: antimicrobial	2.34 ± 0.32^a^	2.29 ± 0.13^a^	2.18 ± 0.08^a^	2.03 ± 0.05^b^	2.26 ± 0.26	2.13 ± 0.26	2.23 ± 0.15	2.13 ± 0.11^*^
Drug resistance: antineoplastic	0.78 ± 0.04^ab^	0.80 ± 0.02^a^	0.78 ± 0.02^ab^	0.76 ± 0.01^b^	0.76 ± 0.06	0.76 ± 0.14	0.76 ± 0.03	0.76 ± 0.03
Endocrine and metabolic disease	0.52 ± 0.06	0.52 ± 0.04	0.49 ± 0.02	0.51 ± 0.01	0.49 ± 0.06	0.48 ± 0.03^*^	0.45 ± 0.05^*^	0.49 ± 0.03^*^
Immune disease	0.16 ± 0.02^a^	0.15 ± 0.01^a^	0.16 ± 0.01^ab^	0.18 ± 0.02^b^	0.13 ± 0.04^AB^	0.13 ± 0.04^AB^	0.12 ± 0.02^A*^	0.15 ± 0.02^B*^
Infectious disease: bacterial	0.67 ± 0.45^a^	0.56 ± 0.08^a^	0.48 ± 0.04^ab^	0.45 ± 0.02^b^	0.56 ± 0.08	0.59 ± 0.11	0.59 ± 0.04^*^	0.52 ± 0.06^*^
Infectious disease: parasitic	0.02 ± 0.03^ab^	0.02 ± 0.01^a^	0.01 ± 00^ab^	0.00 ± 0.01^b^	0.03 ± 0.02^AB^	0.05 ± 0.01^B*^	0.02 ± 0.00^A*^	0.02 ± 0.01^A*^
Infectious disease: viral	0.02 ± 0.01	0.03 ± 0.01	0.02 ± 0.00	0.02 ± 0.00	0.04 ± 0.03^AB^	0.07 ± 0.03^B*^	0.02 ± 0.00^A^	0.02 ± 0.01^A^
Neurodegenerative disease	0.10 ± 0.05^ab^	0.10 ± 0.02^ab^	0.12 ± 0.02^a^	0.08 ± 0.01^b^	0.19 ± 0.08^A*^	0.25 ± 0.06^C*^	0.20 ± 0.03^A*^	0.13 ± 0.04^B*^
Substance dependence	0.00 ± 0.00	0.00 ± 0.00	0.00 ± 0.00	0.00 ± 0.00	0.00 ± 0.00	0.00 ± 0.00	0.00 ± 0.00	0.00 ± 0.00
Environmental Information Processing								
Membrane transport	1.89 ± 0.69^ab^	1.92 ± 0.30^a^	1.54 ± 0.19^b^	1.09 ± 0.09^c^	1.70 ± 0.36^AB^	1.79 ± 0.31^AB^	2.05 ± 0.23^A*^	1.58 ± 0.34^B*^
Signal transduction	0.88 ± 0.13^a^	0.90 ± 0.07^a^	0.84 ± 0.05^a^	0.74 ± 0.04^b^	0.93 ± 0.12^AB^	0.96 ± 0.06^B^	1.05 ± 0.08^C*^	0.86 ± 0.09^A*^
Signaling molecules and interaction	0.00 ± 0.00	0.00 ± 0.00	0.00 ± 0.00	0.00 ± 0.00	0.00 ± 0.00	0.00 ± 0.00	0.00 ± 0.00	0.00 ± 0.00
Organismal Systems								
Aging	0.42 ± 0.04^a^	0.43 ± 0.03^a^	0.47 ± 0.01^b^	0.48 ± 0.02^b^	0.50 ± 0.05^AB*^	0.52 ± 0.07^B^	0.48 ± 0.03^A^	0.48 ± 0.03^A^
Circulatory system	0.00 ± 0.00	0.00 ± 0.00	0.00 ± 0.00	0.00 ± 0.00	0.00 ± 0.00	0.00 ± 0.00	00.00 ± 0.00	00.00 ± 0.00
Development and regeneration	0.00 ± 0.00	0.00 ± 0.00	0.00 ± 0.00	0.00 ± 0.00	0.00 ± 0.00	0.00 ± 0.00	0.00 ± 0.00	0.00 ± 0.00
Digestive system	0.11 ± 0.07^ab^	0.12 ± 0.02^b^	0.06 ± 0.01^a^	0.08 ± 0.01^ab^	0.09 ± 0.03^AB^	0.09 ± 0.040^AB^	0.07 ± 0.03^A^	0.10 ± 0.03^B*^
Endocrine system	0.85 ± 0.10^a^	0.85 ± 0.07^a^	0.76 ± 0.02^b^	0.79 ± 0.02^b^	0.85 ± 0.09^BC^	0.91 ± 0.02^C*^	0.71 ± 0.06^A*^	0.79 ± 0.06^B^
Environmental adaptation	0.20 ± 0.02^ab^	0.21 ± 0.01^a^	0.20 ± 0.01^ab^	0.19 ± 0.01^b^	0.19 ± 0.03^B^	0.19 ± 0.01^B*^	0.22 ± 0.01^A*^	0.21 ± 0.02^AB*^
Excretory system	0.16 ± 0.09^a^	0.14 ± 0.04^a^	0.18 ± 0.03^a^	0.27 ± 0.03^b^	0.17 ± 0.09^A^	0.17 ± 0.05^A^	0.08 ± 0.03^B*^	0.19 ± 0.05^A*^
Immune system	0.29 ± 0.05^a^	0.29 ± 0.01^a^	0.29 ± 0.01^a^	0.27 ± 0.01^b^	0.29 ± 0.05^AB^	0.27 ± 0.06^B^	0.32 ± 0.01^A*^	0.30 ± 0.02^AB*^
Nervous system	0.30 ± 0.05^a^	0.32 ± 0.03^a^	0.32 ± 0.01^ab^	0.35 ± 0.01^b^	0.27 ± 0.04^A^	0.22 ± 0.02^B*^	0.27 ± 0.02^A*^	0.33 ± 0.03^C^
Sensory system	0.00 ± 0.00	0.00 ± 0.00	0.00 ± 0.00	0.00 ± 0.00	0.00 ± 0.00	0.00 ± 0.00	0.00 ± 0.00	0.00 ± 0.00

1. Lowercase letters, such as a, b, c, are used to indicate significant difference in the relative abundance of functional genes of the microbiota among different intestinal segments in the CK group. Capital letters, such as A, B, C, are used to indicate significant difference in the relative abundance of functional genes of the microbiota among different intestinal segments in NAFLD (NA) group. The presence of identical letters in the superscript of the relative abundance of functional genes indicates no significant differences between groups (*p* > 0.05), while the absence of identical letters indicates significant differences between groups (*p* < 0.05). 2. “*” and “**” indicates the significant difference in the relative abundance of functional genes in the same intestinal microbiota between CK group and NAFLD (NA) group. *p < 0.05; **p < 0.01.

## Discussion

There have been advances in the study of gut microbes in human health and disease, but all have been inferred from feces, which are easy to sample and are rich in microbes. The cecum is often used as the research focus in small animals, especially rats and mice. It is the fermentation site of undigested food in mice (Jiminez et al., 2015), whereas in humans, the cecum is much smaller, and fermentation occurs in the colon (He et al., 2019). Alteration of the intestinal microbiota of people suffering from obesity (Zhang et al., 2015; Chambers et al., 2019; Sergeev et al., 2020) or NAFLD (Del Chierico et al., 2016; Hrncir et al., 2021) has been recorded. Studies have found that the relative abundance of Escherichia, Dorea, and Peptoniphilus reportedly increased for the gut microbiota of NAFLD patients. At the same time, that of *Anaerosporobacter*, *Coprococcus*, *Eubacterium*, *Faecalibacterium,* and *Prevotella* decreased (Aron-Wisnewsky et al., 2020). Although these changes in the abundance of microbiota in NAFLD patients and NAFLD mice were not consistent, there were still changes in gut bacteria at all taxonomic levels in human or animal models of obesity, fatty liver, and diabetes. Previous studies showed that the relative abundance of *Firmicutes* and *Proteobacteria* increased, while that of *Bacteroidetes* and *Verrucomicrobia* decreased after a high-fat diet in mice (Walker et al., 2014; Araujo et al., 2017; Wang et al., 2018). All these studies used the cecum or feces as subjects.

Our experiment aims to analyze the differences between the cecal and fecal microbiota of NAFLD mice and other intestinal segments and to investigate whether it would be more appropriate to analyze changes in fecal or cecal microbiota instead of whole intestinal microbiota under NAFLD conditions. Consider that at the time of sampling, mice do not necessarily defecate. Since the mouse colon is highly similar to both fecal microbial (Suzuki and Nachman, 2016; Lkhagva et al., 2021) and metabolite (Zeng et al., 2015) composition, we used distal colonic contents (formed fecal balls) instead of feces. Furthermore, the duodenum had too little content to be detected by high-throughput sequencing. Thus only the jejunum, ileum, cecum, and colon were considered in this study.

In our study, serum levels of TG, TC, HDL and LDL in NAFLD mice were significantly increased compared with CK mice. One study reported that *Actinobacteria* was negatively correlated with TC in mouse fecal microorganisms at the phylum level (Li et al., 2019). Among jejunal microorganisms, *Erysipelotrichaceae* and *Lactobacillaceae* were negatively correlated with TC at the family level (Wang et al., 2021). A study on rats reported that fecal microorganisms *Akkermansia* and *Lactobacillus* were significantly positively associated with TG, TC, and LDL-C and negatively correlated with HDL-C at the genus level (Li R. et al., 2020). In addition, some studies dive into the species level, *Lactobacillus gasseri* and *Lactobacillus taiwanensis* of the fecal microbiota were significantly positively correlated with lipid droplets in the liver (Zeng et al., 2013) and *Desulfovibrio vulgaris* can reduce hepatocyte steatosis and triglyceride accumulation in hepatocytes and liver index (Hong et al., 2021). This indicates intestinal microbiota significantly correlates with serum lipid levels and hepatocyte lesions in NAFLD mice.

At present, research on jejunal microbiota is very rare and limited. Our study found that the alpha diversity (Figure 3) and beta diversity (Figures 4A,B) of the jejunal microbiota of NAFLD mice were not significantly different from those of CK mice, indicating no significant change in the structure of jejunal microbiota in NAFLD mice after a high-fat diet. In NAFLD mice, the beta diversity in the other three intestinal segments (ileum, cecum, and colon; Figure 4), as well as the alpha diversity in the ileum and colon (Figure 3), were significantly changed. Although, as compared to CK mice, the relative abundance of *Peptostreptococcaceae*, *Romboutsia*, and *Allobaculum* in the jejunum of NAFLD mice had altered considerably, functional prediction analysis showed that the relative abundance of primary functional genes had not changed. Only the relative abundance of two secondary active genes (aging and Cardiovascular disease) had significant changes. This indicates that the changes in the microbiota in the jejunum and the other three intestinal segments are inconsistent. We speculated that jejunal microbiota diversity and composition did not change after a high-fat diet mainly because the jejunum had a strong emptying ability; thus, it remained empty for a long time and had the lowest bacterial abundance. A load of microbial community was estimated to be between 10^4^ and 10^7^ CFU/ml (Martinez-Guryn et al., 2019). Compared to other intestinal segments, the lumen of the jejunum is more acidic, with a faster transit time and higher gradients of oxygen, antimicrobials (sIgA and AMPs) and bile acids. Conjugated bile acids, probably together with fatty acids, inhibit bacterial growth directly through their pharmacological properties and signaling properties (Wang et al., 2008; Jia et al., 2018; Lema et al., 2020).

From the limited relevant studies in humans, in general, the ileum serves as a major absorption site for nutrients (e.g., B vitamins and residual nutrients that are not absorbed proximal) and reuptake of BA into the liver circulation, with the latter being more influenced by gut microbes (Martinez-Guryn et al., 2019). *Firmicutes* (*Lactobacillus*, *Veillonella*, *Enterococcus*, and *Clostridium*) and *Proteobacteria* (*Enterobacteria*) are the main bacteria in the ileum (Lema et al., 2020). However, some studies suggest that the ileum microbiota appears to be mainly composed of *Bacteroides*, *Clostridium*, *Enterobacteria*, *Lactobacillus*, and *Veillonella* (Martinez-Guryn et al., 2019), while the dominant bacterial phyla in the colon are *Bacteroidetes* (*Bacteroidaceae*, *Prevotellaceae*, etc.) and *Firmicutes* (*Lachnospiraceae* and *Ruminococcaceae*; Lema et al., 2020). Stefania Vaga et al. studied the mucosal flora of five healthy adults from Stockholm. They found that the two phyla with the highest relative abundance in the ileum were *Firmicutes* and *Bacteroides*, consistent with our mouse flora study (Vaga et al., 2020). There was also a study in mice that reported a significant increase in the relative abundance of *Faecalibaculum* in the ileal microbiota of HFD-induced NAFLD mice compared with normal mice (Mu et al., 2020). Our study found that alpha and beta diversity in the ileum of NAFLD mice changed after a high-fat diet, but alpha diversity in the cecum did not change. The *Rank Sum test* results showed that the relative abundance of Firmicutes and Proteobacteria in the ileum did not change after a high-fat diet but increased significantly in the cecum and colon.

Meanwhile, through *rank sum test* and Lefse analysis, it was found that the change trend of characteristic flora and main bacterial composition of ileum was different from that of cecum and colon. Therefore, the changes in the ileum of NAFLD mice were different from those in the large intestine (cecum and colon). The main reason is that the ileum is vastly different from the large intestine in terms of bacterial abundance, diversity, and flora composition. The microbial load in the ileum, estimated at 10^3^ ~ 10^8^ CFU/ml, contains both the jejunal microbiota and the large intestine microbiota and is a transition zone between the sparse populations of aerobic microbiota of the jejunum and the very dense populations of strict anaerobic in the colon (Rao and Bhagatwala, 2019). In our study, the number of OTUs in the ileum of CK mice was the largest (Figures 6B,E), which was mainly composed of facultative and obligate anaerobic bacteria (Rao and Bhagatwala, 2019; Shahir et al., 2020; van Kessel et al., 2020). Facultative anaerobic bacteria included *Proteobacteria* and *Lactobacillaceae*, which are equipped to resist the synergistic effect of bile acids and antimicrobial peptides (Tropini et al., 2017).

Additionally, our study found that there were no differences in both alpha and beta diversity between a normal colon and cecum, as well as the relative abundance of major bacteria at phylum, family, and genus levels, indicating that the microbiota of the colon and cecum were highly similar and that the microbiota of both could be substituted for each other during microbiota research and fecal microbiota transplantation. Interestingly, after a high-fat diet, the characteristics of the changes in the abundance of major bacteria were very similar. The functional prediction of intestinal microbiota also showed that the relative abundance of primary and secondary functional genes in cecum and colon was similar. This may be due to the short colon of the mouse, which mainly plays the function of absorbing water and electrolytes as well as transport, and the main fermentation has taken place in the cecum (Brown et al., 2018). However, there are differences in the structure and function of the cecum and colon between humans and mice. In animals, fermentation occurs mainly in the cecum (Brown et al., 2018), while in humans, it occurs primarily in the colon. Human colon microbiota has been studied in greater depth than cecal microbiota. The colon has greater microbial abundance and diversity than the small intestine (jejunum and ileum). Despite its shorter length, food stays in the colon (10 h to several days) longer than it does in the small intestine (1–5 h; Zhuang et al., 2020). The thicker inner and outer layers of mucus (Hansson, 2012) are important barriers to bacteria (an estimated 10^10^ to 10^12^ CFU/ml, including *Firmicutes*, *Bacteroidetes* and other phyla; Jakobsson et al., 2015; Martinez-Guryn et al., 2019). In the colon, the relative abundance of *Butyricicoccus*, *Allobaculum*, *Alloprevotella*, *Lachnospiraceae NK4A136 group*, *Parasutterella*, and *uncultured bacterium Muribaculaceae* decreased, while the relative abundance of *Campylobacter*, *Dubosiella*, *Faecalibaculum* and *Fusobacterium* increased (Mu et al., 2020). In our study, *Bacteroidetes* was the main bacteria in the colon of CK mice, with a relative abundance of 72.07%, followed by *Firmicutes* with a relative abundance of 15.36%, and *Verrucomicrobia* with a relative abundance of 8.67% (Supplementary Table S2). At the family level, the bacteria with the highest relative abundance were *Muribaculaceae*, *Akkermansiaceae*, *Lachnospiaceae*, *Erysipelotrichaceae*, and *Lactobacillaeae* (Supplementary Table S3). At the genus level, the five genera with the highest relative abundance were *Akkermansia*, *Allobaculum*, *Muribaculumg*, *Lactobacillus*, and *Parabacteroides* (Supplementary Table S4). Compared with the results of human related studies, was found that in the colon, human and mice have similar bacterial species at the phylum and family level, but have differences at the genus level, which is likely caused by host species. Two studies on cecal microbiota in mice showed a significant increase in the abundance of *Faecalibaculum* in cecal microbiota in NAFLD mice compared with normal mice (Mu et al., 2020; Gu et al., 2022), which was similar to our results.

## Conclusion

In conclusion, the structure of jejunal microbiota in NAFLD mice induced by a high-fat diet did not change significantly. In contrast, the intestinal microbiota of the ileum, cecum, and colon (feces) changed significantly. However, the characteristics of the changes in the ileum were substantially different from that of the cecum and colon (feces), and the changes in the cecum and colon (feces) were similar. Therefore, the study results of cecal and colonic (fecal) microbiota cannot completely represent the results of jejunal and ileal microbiota. This is of reference significance for future studies on the role of intestinal microbiota in NAFLD.

## Data availability statement

The data presented in the study are deposited in the NCBI-SRA repository (https://www.ncbi.nlm.nih.gov/bioproject/PRJNA877487), accession number PRJNA877487.

## Ethics statement

The animal study was reviewed and approved by the Animal Ethics Committee of Southwest Medical University (No. of Animal Ethics Approval: SWMU2019463).

## Author contributions

HY and CG: conceptualization. SbL, YW, JrH, BC, WX, and FZ: data curation. YL, BC, SyL, and FK: formal analysis. GY and CG: funding acquisition. SyL, YL, LC, LH, MH, QY, WX, and JhH: investigation. GY, ZY, SY, HY, and CG: methodology. CG: project administration. YL and JrH: resources. ZY and CG: supervision. QY, HY, and CG: validation. YW: visualization. GY, SbL, YW, and BC: writing - original draft. SbL, LH, MH, YT, WL, SY, BA, HY, and CG: writing - review and editing. All authors have read and agreed to the published version of the manuscript.

## Funding

This research was funded by the “Applied Basic Research Program of Science and Technology Department of Sichuan Province (2020YJ0399), the Liangshan Science and Technology Bureau (20ZDYF0070), PhD Project of Xichang University (LGLZ201809), and Luzhou Municipal People’s Government – Southwest Medical University Technology Strategy Project (2020LZXNYDJ20).”

## Conflict of interest

The authors declare that the research was conducted in the absence of any commercial or financial relationships that could be construed as a potential conflict of interest.

## Publisher’s note

All claims expressed in this article are solely those of the authors and do not necessarily represent those of their affiliated organizations, or those of the publisher, the editors and the reviewers. Any product that may be evaluated in this article, or claim that may be made by its manufacturer, is not guaranteed or endorsed by the publisher.

## Footnotes

^1^FLASH [Online]. Available: http://ccb.jhu.edu/software/FLASH/ (Accessed 2019).

^2^Qiime [Online]. Available: http://qiime.org/scripts/split_libraries.html (Accessed 2019).

^3^UCHIME [Online]. Available: http://drive5.com/uchime/uchime_download.html (Accessed 2019).

^4^Uparse [Online]. Available: http://drive5.com/uparse/ (Accessed 2019).

^5^Mothur [Online]. Available: https://mothur.org/CITATION/ (Accessed 2019).

^6^Picrust [Online]. Available: https://github.com/picrust (Accessed 2019).

## Supplementary material

SUPPLEMENTARY FIGURE S1 Relative abundance of bacteria at family level. **(A)** Stacked column graph of the top 10 families with the highest relative abundance. **(B)** The box diagram was used to analyze the differences in relative abundance of the five families with the highest relative abundance. *Wilcox Rank-sum test* was used to compare the significance of the same intestinal segment between the CK group and the NAFLD group; *Kruskal-wallis test* was used to compare the differences between different intestinal segments in the same group. *indicate significant difference in the different intestinal segments in the CK group and NAFLD group. ^#^indicates the significant difference in the same intestinal segments between CK group and NAFLD group. *or^#^ *p* < 0.05; **or^##^ *p* < 0.01.
