# Supplementary figures and images for: Characteristics of intestinal microbiota in C57BL/6 mice with non-alcoholic fatty liver induced by high-fat diet
Source: Front Microbiol. 2022 Dec 22;13:1051200. doi: 10.3389/fmicb.2022.1051200 (PMC9813237; doi:10.3389/fmicb.2022.1051200)

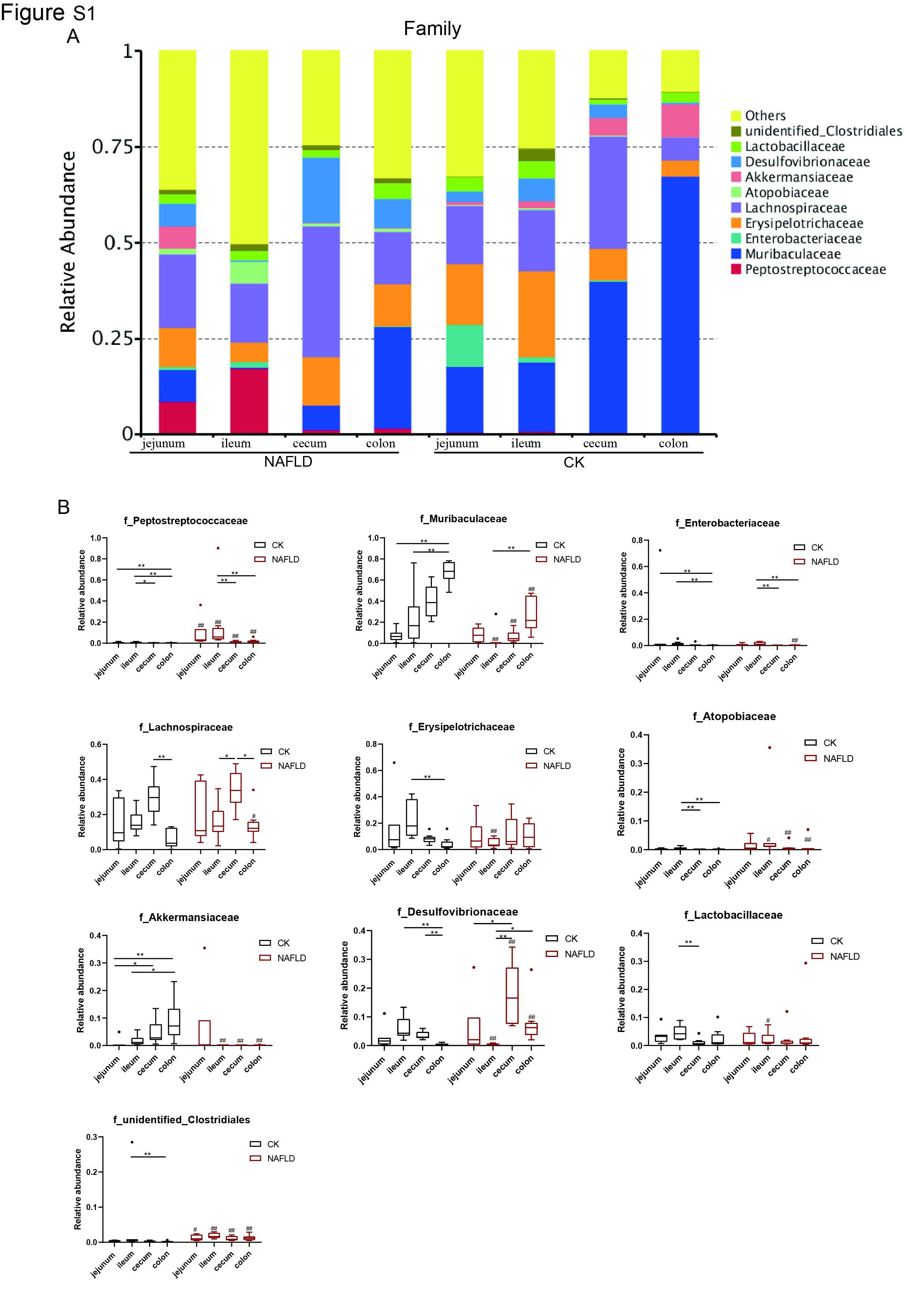

Supplement: SUPPLEMENTARY FIGURE S1 — Relative abundance of bacteria at family level. (A) Stacked column graph of the top 10 families with the highest relative abundance. (B) The box diagram was used to analyze the differences in relative abundance of the five families with the highest relative abundance. Wilcox Rank-sum test was used to compare the significance of the same intestinal segment between the CK group and the NAFLD group; Kruskal-wallis test was used to compare the differences between different intestinal segments in the same group. *indicate significant difference in the different intestinal segments in the CK group and NAFLD group. #indicates the significant difference in the same intestinal segments between CK group and NAFLD group. *or# p < 0.05; **or## p < 0.01. [file Image_1.JPEG]
